# Amination of Nitroazoles — A Comparative Study of Structural and Energetic Properties

**DOI:** 10.3390/molecules19010896

**Published:** 2014-01-14

**Authors:** Xiuxiu Zhao, Cai Qi, Lubo Zhang, Yuan Wang, Shenghua Li, Fengqi Zhao, Siping Pang

**Affiliations:** 1 State Key Laboratory of Explosion Science and Technology, School of Material Science & Engineering, Beijing Institute of Technology, Beijing 100081, China; E-Mails: zhaoxiuxiu010@163.com (X.Z.); qicaijingbei@163.com (C.Q.); xiaoahexiaob@163.com (L.Z.); invincibly@yeah.net (Y.W.); 2 Burning key laboratory, Xi’an Modern Chemistry Research Institute, Xi’an 710065, China; E-Mail: zhaofqi@163.com

**Keywords:** energetic materials, detonation properties, azole-based compound, *C*-amino group, *N*-amino group

## Abstract

In this work, 3-nitro-1*H*-1,2,4-triazole (**1**) and 3,5-dinitro-1*H*-pyrazole (**2**) were *C*-aminated and *N*-aminated using different amination agents, yielding their respective *C*-amino and *N*-amino products. All compounds were fully characterized by NMR (^1^H, ^13^C, ^15^N), IR spectroscopy, differential scanning calorimetry (DSC). X-ray crystallographic measurements were performed and delivered insight into structural characteristics as well as inter- and intramolecular interactions of the products. Their impact sensitivities were measured by using standard BAM fallhammer techniques and their explosive performances were computed using the EXPLO 5.05 program. A comparative study on the influence of those different amino substituents on the structural and energetic properties (such as density, stability, heat of formation, detonation performance) is presented. The results showed that the incorporation of an *N*-amino group into a nitroazole ring can improve nitrogen content, heat of formation and impact sensitivity, while the introduction of a *C*-amino group can enhance density, detonation velocity and pressure. The potential of *N*-amino and *C*-amino moieties for the design of next generation energetic materials is explored.

## 1. Introduction

Energetic materials are extensively used for a variety of military purposes and industrial applications. The synthesis and development of new energetic materials with higher performance and lower sensitivity towards heat, shock, friction and electrostatic discharge has attracted considerable recent interest [1]. However, the requirements of insensitivity and high energy are often mutually exclusive, which makes the development of new high-energy materials an interesting but challenging task. In the pursuit of new energetic materials, nitrogen-rich heterocyclic compounds have attracted considerable attention [2,3,4,5,6]. A prominent family of novel high-energy-density materials are azole-based compounds since they are generally highly endothermic with high densities and low sensitivities towards external stimuli [7,8]. Recently, to obtain better detonation properties, azole rings have often been modified using energy-rich functional groups like amino (-NH_2_) [9,10,11], nitro (-NO_2_) [12,13,14,15,16,17,18,19], nitroamine (-NNO_2_) [20,21,22] and azido (-N_3_) [23,24]. The introduction of a nitro group improves the oxygen balance and density of energetic compounds, and thus the detonation properties. However, highly nitrated azole-based compounds usually suffer from several prohibitive drawbacks including lower stability and greater sensitivity due to their high acidities [25,26,27]. In contrast, the introduction of an amino group is a more effective method for enhancing the stability and lowering the sensitivity of energetic materials. Moreover, the amino group can undergo further functionalization, such as nitration [20,21,22,28], diazotization [29,30,31], or trinitroethylation [32,33] to provide versatile energetic materials.

**Figure 1:**
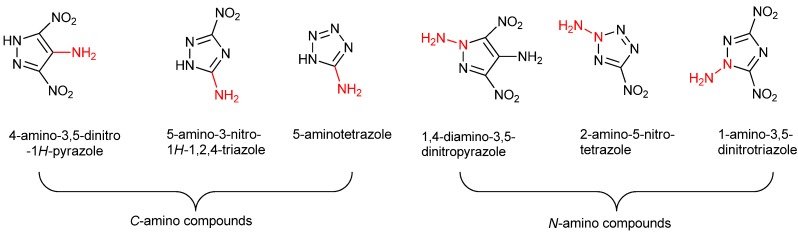
Two different connection modes of amino groups in azole-based compounds.

Generally, amino groups exhibit two different connection modes in azole-based compounds (Figure 1). The first mode is where the amino group is bonded to the carbon atom of the azole ring (*C*-amino) [12]. 5-Amino-3-nitro-1*H*-1,2,4-triazole [34,35], 4-amino-3,5-dinitro-1*H*-pyrazole [36], 5-aminotetrazole [37,38,39] are three representative compounds of this kind. Another is where the amino group is attached to the nitrogen atom of the azole ring (*N*-amino) [13], such as in 2-amino-5-nitrotetrazole [40], 1-amino-3,5-dinitro-1,2,4-triazole [41], and 1,4-diamino-3,5-dinitropyrazole [42].

Different kinds of amino groups may influence the performance of azole-based compounds. Although a few azole-based compounds containing different amino groups have been reported, the systematic study of the effect of different amino groups is surprisingly quite scarce. However, understanding the basic principles of structure-property relationships of different amino compounds is highly desirable, as such an understanding would allow for a more rational design process to yield the desired properties.

Owing to their high heats of formation and ring strain, nitroazoles (nitropyrazoles, nitrotriazoles and nitrotetrazoles) have been used for the preparation of high performance primary and secondary explosives [12,13,14,15,16,17,18,19]. Nitrotetrazole rings (such as 5-nitrotetrazole) cannot allow further *C*-amination due to the absence of an additional carbon atom. In order to develop new azole-based energetic compounds and to explore the influence of those different amino substituents on the structural and energetic properties, we have selected 3-nitro-1*H*-1,2,4-triazole (**1**) and 3,5-dinitro-1*H*-pyrazole (**2**) as substrates because they possess high nitrogen content, good thermal stabilities, and have additional nitrogen and carbon atoms in the azole ring amenable to further *C*-amination and *N*-amination reactions. Herein, we report the synthesis of *C*-aminonitroazoles [5-amino-3-nitro-1H-1,2,4-triazole (**1c**), 4-amino-3,5-dinitro-1H-pyrazole (**2c**)) and the corresponding N-aminonitroazoles (1-amino-3-nitro-1,2,4-triazole (**1n**) and 1-amino-3,5-dinitro-pyrazole (**2n**)]. The compound **1n** has been reported as a reactant toward well-characterized products in previous publications, but neither of those gave any experimental details of its preparation and energetic properties [43]. We synthesized **1n**, using hydroxylamine-*O*-sulfonic acid (HOSA) as the amination reagent, and fully characterized it. Though both on structural and energetic properties of compounds **1**, **1c**, **2** and **2c** were characterized in previous literature reports [34,35,36,44,45], the focus of this contribution is a comparative study on the influence of those different amino substituents on the structural and energetic properties. Thus, for the sake of comparison, it was necessary to fully characterize these selected nitroazoles containing *C*-amino or *N*-amino groups and their energetic properties using the same methods and standards. In addition, a simple and straightforward synthetic pathway to **1c** was also developed. The potential of *N*-amino and *C*-amino moieties for the design of next generation energetic materials will be evaluated.

## 2. Results and Discussion

### 2.1. Synthesis

**Scheme 1:**
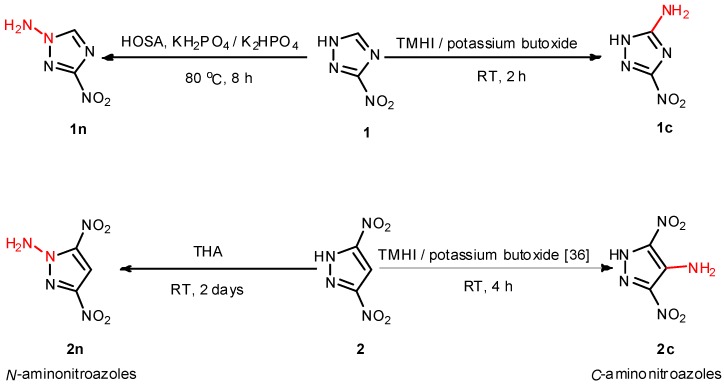
Synthesis of *C*-aminonitroazoles and *N*-aminonitrozoles **1c**, **2c** and **1n**, **2n**.

The synthetic pathways to compounds **1c**, **2c**, **1n** and **2n** are depicted in Scheme 1. *C*-aminonitroazole **2c** was prepared by one-step *C*-amination reaction of 3,5-dinitro-1*H*-pyrazole with 1,1,1-trimethyhydrazinium iodide (TMHI), according to the literature procedure [36]. In contrast, **1c** was previously synthesized as an important intermediate for energetic materials [18,19] by a three-step synthetic route [34,46,47,48]. The route involves the diazotization of 3,5-diaminotriazole with sodium nitrite in sulfuric acid, following reaction with ammonium carbonate, and reduction with hydrazine hydrate. The reaction pathway did not seem to be operationally simple or safe because the reaction sequence was too long and tedious, and the highly sensitive and volatile diazonium salt was used. Besides the solubility of the intermediate 3,5-dinitro-1*H*-1,2,4-triazole in diethyl ether was poor, making it time-consuming and inefficient to extract 3,5-dinitro-1*H*-1,2,4-triazole using diethyl ether [49,50,51]. In our improved synthetic pathway, compound **1c** was successfully obtained by one-step *C*-amination reaction of 3-nitro-1*H*-1,2,4-triazole with TMHI. In comparison with previous synthetic routes of **1c**, it was preferable to employ one-step amination reaction because the synthetic route was simple and straightforward, the starting material was readily available and inexpensive, and equally importantly, the yield was higher.

HOSA is well-known as an efficient reagent for the *N*-amination of electron-rich heterocycles. However, HOSA *N*-amination is difficult to extend to electron-poor systems [40]. The possible reason is that the hydrolysis rate of HOSA is faster than that of the amination reaction in the alkali solution [52]. In the case of **1n**, we found that when a buffered solution of KH_2_PO_4_/K_2_HPO_4_ was used as solvent, the electron-poor compound 3-nitro-1*H*-1,2,4-triazole was successfully aminated to give the corresponding product **1n**. However, an attempt to aminate ammonium 3,5-dinitro-1*H*-pyrazolate with HOSA under the same reaction condition was unsuccessful. In this case, the more powerful amination agent O-tosylhydroxylamine (THA) afforded the desired product **2n**.

### 2.2. Spectroscopy

All compounds **1c**, **2c**, **1n** and **2n** were characterized using IR and ^1^H-, ^13^C- and ^15^N-NMR spectroscopy**,** as well as elemental analysis. The IR spectra of compounds **1c**, **1n**, **2c** and **2n** showed several strong absorption bands at approximately 1,587, 1,511, 1,437, 1,330 and 1,185 cm^−1^, which were attributed to the C-NO_2_ and C=N bonds. In addition, the intense absorption bands in the range of 3,100–3,500 cm^−1^ were assigned to the N-H bonds.

In the ^1^H-NMR spectra, the *C*-amino proton resonances were observed at 6.81 (**1c**) and 7.13 (**2c**) ppm, while the proton signals of the *N*-amino groups were found at 7.16 (**1n**) and 7.88 ppm (**2n**), which were shifted downshield compared to those of the *C*-amino groups. In contrast, the *N*-amino proton signal of **1n** appeared at higher field than that of its analogous *N*-amino compound 1-amino-3,5-dinitro-1,2,4-triazole (δ = 8.26 ppm) [40].

**Figure 2:**
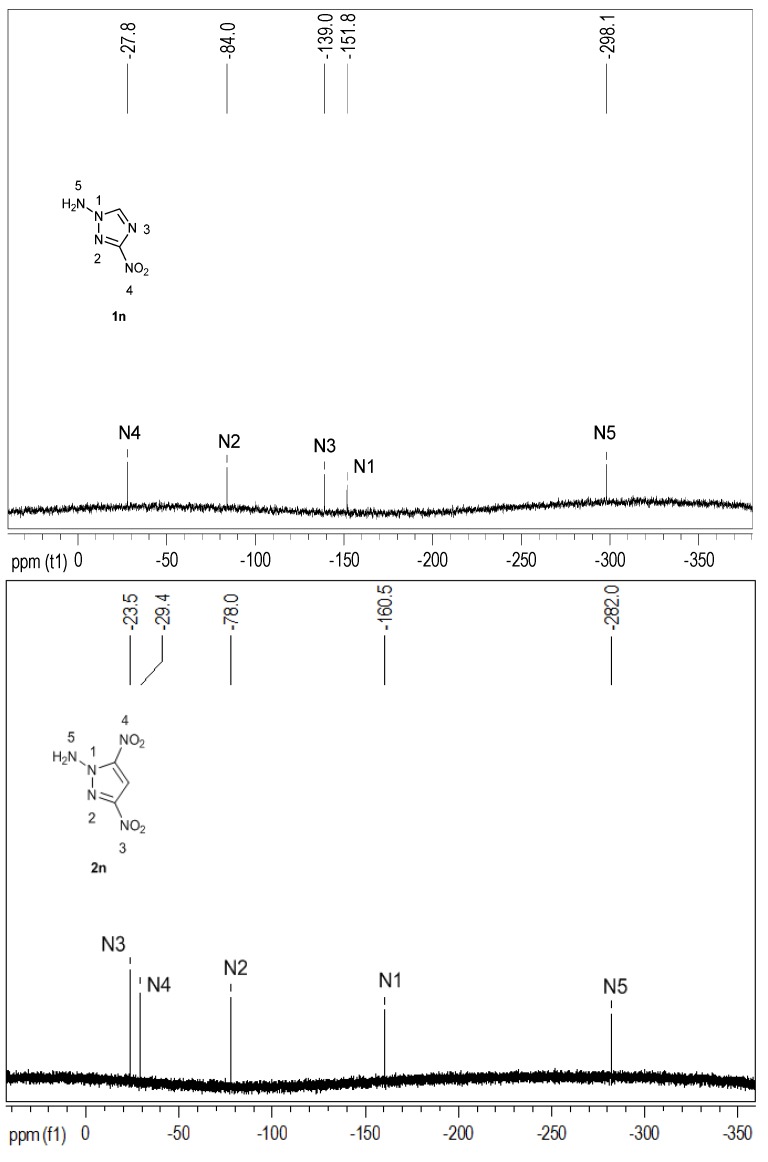
^15^N-NMR spectra of **1n** and **2n**.

Figure 2 shows the ^15^N-NMR spectra of **1n** and **2n**. The chemical shifts were given with respect to CH_3_NO_2_ as an external standard. The ^15^N-NMR spectrum of **1n** had five signals at δ = −298.1, −151.8, −139.0, −84.0 and −7.8 ppm. The signal for the *N*-amino group appeared at the highest field. The chemical shifts of N1, N2 and N3 of the triazole ring as well as the nitro group were found in the expected range similar to those of the recently published 3,3'-dinitro-5,5'-bi-1*H*-1,2,4-triazole [53]. Moreover, in the ^15^N-NMR spectrum of **2n,** five well resolved resonances at δ = −282.0, −160.5, −78.0, −29.4 and −23.5 ppm, can also be observed. The chemical shifts of the pyrazole nitrogen atoms N1 and N2 agreed with those of **2c**. The largest difference in comparison to **2c** can be observed for the nitrogen atom (N5) of the amino group, the signal in **2n** was now located at a chemical shift of −282.0 ppm, whereas the signal of the *C*-amino group of **2c** appeared at −316.0 ppm.

### 2.3. Crystal Structure

In the following, the structural properties of **1n** and **2n** will be discussed in detail to point out the structural characteristics of *N*-aminonitroazoles in comparison to the corresponding *C*-aminonitroazoles. The X-ray crystallographic data for all compounds were collected by a Rigaku RAXIS IP diffractometer using Mok*α* (λ = 0.71073 Å) and integrated with the SHELXTL crystallographic software package. The structure was solved by direct methods with SHELXS-97 and expanded by using the Fourier technique. The non-hydrogen atoms were refined anisotropically; hydrogen atoms were located in a difference Fourier map. Selected crystallographic data for **1n** and **2n** were compiled in Table S3. A comparison of selected bond lengths and bond angles for compounds **1n** and **2n** with **1c** and **2c** was given in Tables S1 and S2.

**Figure 3:**
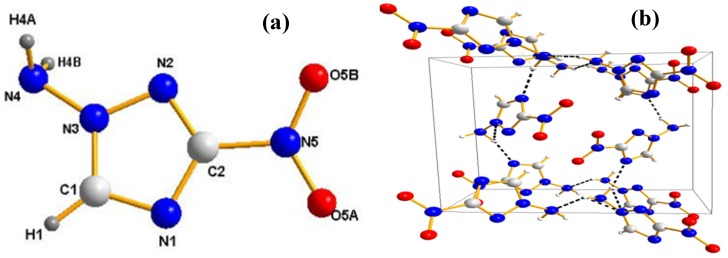
(**a**) Molecular unit of compound **1n**, which shows the labeling scheme; ellipsoids are set at 50% probability. (**b**) Intermolecular interactions in the crystal structure of **1n** (view along c-axis), hydrogen bonds are marked as dotted lines.

Compound **1n** crystallizes in the orthorhombic space group *P*na2_1_. The structure is shown in Figure 3. As shown in this figure, the triazole ring of **1n** still shows planar geometry due to aromaticity. The two hydrogen atoms of the amino group project on opposite sides of the plane defined by the triazole ring. The nitro group is almost coplanar with the triazole ring. In comparison to the crystallographically determined density of **1c** (C2/c, 1.82 g·cm^−3^), **1n** has a relatively lower density (1.70 g·cm^−3^). Due to the increased amount of hydrogen bonding in these compounds *C*-aminonitroazoles have significantly higher densities than the ones observed for the corresponding *N*-aminonitroazoles. In the crystal structure of **1c** [44], each molecule is surrounded by four adjacent molecules via six strong hydrogen bonds toward the nitrogen atoms of *C*-amino group and the triazole ring, and the oxygen atoms of the nitro group. It is remarkable that all nitrogen atoms of the triazole ring act as acceptor atoms for hydrogen bonds. The extensive intermolecular hydrogen bonds form a complex 3D network which contributes greatly to the higher density. In contrast, the crystal structure of **1n** contains only one individual hydrogen bond (N4-H4B…N1, 3.006 Å). Unfortunately, the oxygen atoms of the nitro group in the molecule do not participate as acceptor atoms in any of the hydrogen bonds (Figure 3B).

**Figure 4:**
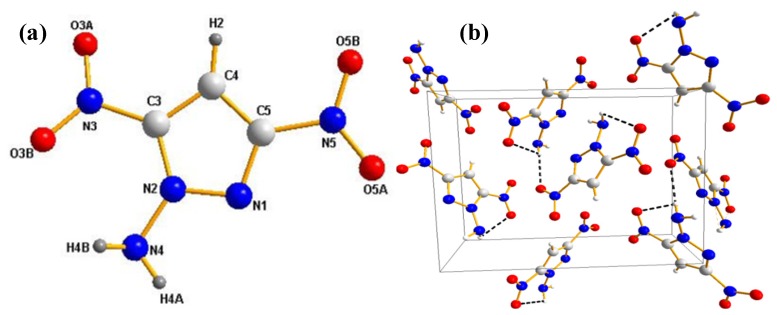
(**a**) Molecular unit of compound **2n**, which shows the labeling scheme; ellipsoids are set at 50% probability. (**b**) Intermolecular interactions in the crystal structure of **2n** (view along c-axis), hydrogen bonds are marked as dotted lines.

Compound **2n** crystallizes in the orthorhombic space group P2_1_2_1_2_1_ with four molecules in the unit cell, which is very similar to the corresponding *C*-aminonitroazole **2c** [36]. The density of **2c** is 1.90 g·cm^−3^ is notable higher than **2n** (1.81 g·cm^−3^). The remarkably high density can also be rationalized in terms of the strong hydrogen bonding network. In the crystal structure, **2c** facilitates a dense packing scheme, which resembles that of TATB insofar as there are two strong intramolecular hydrogen bonds within each molecule and six strong intermolecular hydrogen bonds around each molecule. However, only two hydrogen bonds are observed in the structure of **2n** (intramolecular hydrogen bond: N4-N4B…O3B, 2.850 Å; intermolecular hydrogen bond: N4'-H4B'…O5A, 3.177 Å) (Figure 4B).

Therefore, since more hydrogen bonds are present in the structures of *C*-aminonitroazoles, their densities are significantly higher than those observed of the corresponding *N*-aminonitroazoles, but all the above aminonitroazoles contain intra- or intermolecular hydrogen bonding, which contribute greatly to low sensitivities.

### 2.4. Physicochemical Properties

Because all the materials studied are energetic compounds, the energetic behavior of all the compounds was investigated (Table 1). In order to perform a more comprehensive study of the effect of different amino groups on energetic properties, 1,4-diamino-3,5-dinitro-pyrazole (**2cn**), 5-nitrotetrazole (**3**), its corresponding *N*-aminonitroazole 2-amino-5-nitrotetrazole (**3n**) were also discussed according to the values reported in the previous literature (Figure 1) [40,42,54].

**Table 1:** Energetic properties and detonation parameters for azole-based compounds, TNT and TATB.

Comp.	N *^a^*	Ω *^b^*	IS *^c^*	T_d_*^d^*	*D ^e^*	HOF *^f^*	P *^g^*	D *^h^*
1	49.12	−42.11	>40	218	1.727	216.9	28.9	8255
1n	54.26	−43.41	>40	137	1.698	217.9	27.4	8194
1c	54.26	−43.41	>40	246(241 *^j^*)	1.819	201.8	31.5	8582
2	35.44	−30.88	>40	295	1.844	128.5	32.7	8388
2n	40.46	−32.37	>40	112	1.810	144.4	31.7	8384
2c	40.46	−32.37	>40	178(177 *^j^*)	1.900	96.3	34.2	8573
2cn	44.68	−34.04	>40	241	1.880	166.0	34.6 (35.0^i^)	8712 (8732 ^i^)
3	60.87	−7.00	>1	130	1.899	281.0	39.2 (39.0^i^)	9156 (9457^i^)
3n	64.61	−12.30	>1	140	1.791	376.4	36.7(36.8 ^i^)	9056 (9087 ^i^)
TNT	18.50	−74.01	15	295	1.650	−67.0	19.5	6881
TATB	32.55	−55.81	50	360	1.930	−154.2	31.2	8114

^a^ Nitrogen content (%). ^b^ Oxygen balance (Ω = (xO − 2yC − 1/2zH) M/1,600) (%). ^c^ Impact sensitivity (BAM drop hammer) (J). ^d^ Decomposition temperature (from DSC, β = 5 °C·min^−1^) (°C). ^e^ Density from X-ray diffraction (g·cm^−3^). ^f^ Calculated enthalpy of formation (kJ·mol^−1^). ^g^ Detonation pressure (GPa). ^h^ Detonation velocity (m·s^−1^). ^i, j^ The values were reported in the previous literatures [34,36,40,42,54].

One of the most important physical properties of a solid energetic material is its density. As shown in Table 1, the densities of *C*-aminonitroazoles are higher than those of the corresponding *N*-aminonitroazoles as a consequence of multiple intermolecular bonding interactions, as exemplified above in the cases of **1c** and **2c**. It is worth pointing out that these *C*-aminonitroazoles and *N*-aminonitroazoles also have high nitrogen content. Some of them exceed 50%, and reach as high as 60%. It is well-known that the amino group helps to enhance nitrogen content.

The thermal stabilities of aminonitroazoles were determined by differential scanning calorimetric (DSC) and thermogravimetric analysis (TG) measurements. Among them, the two *N*-amino compounds **1n** and **2n** melt with decomposition. In comparison to the parent relatives, the decomposition temperatures of **1n** (T_d_ = 137 °C) and **2n** (T_d_ = 112 °C) are much lower. Surprisingly, when extended to nitrotetrazole ring, it is observed that **3n** (T_d_ = 140 °C) is a little higher than **3** (T_d_ = 130 °C). Furthermore, for *C*-amino compounds, the decomposition temperatures of **1c** (T_d_ = 246 °C) is higher than that of **1** (T_d_ = 218 °C), while **2c** (T_d_ = 178 °C) is lower than that of **2** (T_d_ = 295 °C). In short, the decomposition temperature of aminonitroazoles did not show a common trend. This is probably related to many factors, such as the stability of the azole ring, the number of substituents and the different effect of electron-withdrawing groups and electron-donation groups at the azole ring [55].

The heat of formation, which is directly related to the number of N-N bonds in the molecule, is another important parameter in evaluating the performance of energetic compounds. As shown in Table 1, most of the compounds exhibit positive heats of formation. Among them, the heats of formation of two new compounds are 217.9 kJ·mol^−1^ (**1n**) and 144.4 (**2n**) kJ·mol^−1^, respectively, which are superior to those of their parent compounds (**1**, 216.9 kJ·mol^−1^; **2**, 128.5 kJ·mol^−1^) because the *N*-amino group can produce the additional energetic N-N bond. Furthermore, *N*-aminonitrotetrazole (**3n**) and *N*-aminonitroazole (**2cn**) also show the same trend. Therefore, introducing N-NH_2_ into a nitrogen-rich heterocycle is an effective way to obtain a higher heat of formation. In contrast, the *C*-amino compounds display an opposite trend if compared to their corresponding parent compounds. The heat of formation of **1c** is 201.8 kJ·mol^−1^, which is lower than that of the starting material **1** (216.9 kJ·mol^−1^). A plausible explanation for this could that in *C*-aminonitroazoles, the *C*-amino moiety could have a slight negative impact on heat of formation.

With the data for molecular weight, density, and heat of formation in hand, the detonation velocities and pressures of all the compounds were calculated using the EXPLO 5.05 program [56]. The calculated detonation pressures lie in the range between 27.4 and 39.2 GPa and the calculated detonation velocities fall in the range between 8,194 and 9,156 m·s^−1^. Of them, *N*-aminonitroazoles **1n** and **2n** are comparable to TATB (31.2 GPa, 8,114 m·s^−1^) and superior to TNT (19.5 GPa, 6,881 m·s^−1^). From Table 1, one trend in the detonation performance can also be observed: the compounds, which contain a *C*-amino group exhibit higher detonation velocities and pressures than their corresponding parents due to possessing higher densities. On the contrary, *N*-aminonitroazoles show relatively lower detonation performance.

Impact sensitivity measurements were made by using standard BAM fallhammer techniques. Most of aminonitroazoles show low sensitivities towards impact such as **1n**, **1c**, **2n**, **2c** and **2cn** (IS > 40 J). Therefore, the introduction of the amino group is favorable for improving impact sensitivity due to considerable intra- or intermolecular hydrogen bonding in these molecules.

## 3. Experimental

### 3.1. General Information

All starting materials were commercially available and used as received. IR spectra were recorded on a Nicolet Magna IR 560 spectrophotometer (KBr pellets, Madison, USA). NMR experiments were performed at room temperature on a Bruker ARX 400 instrument (Zurich, Switzerland) with TMS as an internal standard. MS EI were recorded on a GCTMS Micromass UK spectrometer (Manchester, UK) and MS ESI were recorded on an Agilent 6120 LCMS spectrometer (Santa Clara, CA, USA). Elemental analysis were performed on an Elementar Vario EL (Bremen, Germany) instrument. Crystal structures were determined on a Rigaku RAXIS IP diffractometer (Rigaku Corporation, Tokyo, Japan) with the SHELXTL crystallographic software package of molecular structure. CCDC 958583 and 958582 contain the supplementary crystallographic data for this paper. These data can be obtained free of charge from The Cambridge Crystallographic Data Centre via http://www.ccdc.cam.ac.uk/data_request/cif. To determine the thermal stability of the described compounds, a TA-DSC Q2000 differential scanning calorimeter (heating rate: 5 °C·min^−1^, New Castle, DE, USA) was used.

***CAUTION**: Although we experienced no problems during the synthesis of the reported compounds, standard safety precautions (leather gloves, face shield and ear plugs) should be used when handling these energetic materials.*

### 3.2. 5-Amino-3-nitro-1H-1,2,4-triazole **(1c)**

5-Nitro-1*H*-1,2,4-triazole (0.257 g, 2.25 mmol) and 1,1,1-trimethylhydrazinium iodide (0.504 g, 2.49 mmol) were dissolved in DMSO (12.0 mL). Solid potassium *t*-butoxide (0.741 g, 6.60 mmol) was then added in one portion while stirring. The clear yellow solution immediately turned to a dark crimson red color as the base dissolved, and the odor of trimethylamine was noted. The reaction mixture was stirred at room temperature for 4 h, after which it was poured onto 12 g of ice and acidified to pH 3 with 10% HCl. The resulting solids were suction filtered, washed with cold water and air dried. Crystallization from ethanol afforded pure **1c** (2.53 g, 85%). DSC (5 °C·min^−1^): 246 °C (T_d_); ^1^H-NMR (DMSO-*d_6_*) δ = 6.81 (s, 2H), 13.15 (s, 1H) ppm; ^13^C-NMR (DMSO-*d_6_*) δ = 157.3 (s, 1C), 160.9 (s, 1C) ppm; MS (ESI) *m/z*: 128 [M-1] ^+^; Elemental analysis calcd (%) for (C_2_H_3_N_5_O_2_): C 18.61, N 54.26, H 2.34; found: C 18.50, N 54.44, H 2.51.

### 3.3. 3,5-Dinitro-1H-pyrazole **2**

According to the literature procedure [48], 3,5-dinitro-1*H*-pyrazole was obtained through rearrangement of 1,3-dinitropyrazole. A solution of 1,3-dinitropyrazole (2 g, 13 mmol) in benzonitrile (30 mL) was heated to 147 °C for 77 h. Then the reaction mixture was treated with ammonia at room temperature. The ammonium salt of 3,5-dinitro-1*H*-pyrazole (2 g, 90%) was obtained. The solution of the ammonium salt of 3,5-dinitro-1*H*-pyrazole (0.9 g, 5 mmol) in water was neutralize with hydrochloric acid, extracted with diethyl ether, dried over magnesium sulfate and the solvent was removed in vacuum. Crystallization from water afforded pure **2** (0.75 g, 92%). DSC (5 °C·min^−1^): 173 °C (T_m_), 295 °C (T_d_); ^1^H-NMR (DMSO-*d_6_*) δ = 7.94 (s, 1H), 14.85 (s, 1H) ppm; MS (EI) *m/z*: 158 (M^+^). Elemental analysis calcd (%) for (C_3_H_2_N_4_O_4_): C 22.79, H 1.28, N 35.44; Found: C 22.67, H 1.31, N 35.61.

### 3.4. 4-Amino-3,5-dinitro-1H-pyrazole **2c**

This compound was prepared according to the literature procedure [36], and the yield was 90%. DSC (5 °C·min^−1^): 178 °C (T_d_); ^1^H-NMR (DMSO-*d_6_*) δ = 7.13 (s, 2H) ppm; MS (EI) *m/z*: 172 (M^+^); Elemental analysis calcd (%) for (C_3_H_3_N_5_O_4_): C 20.81, H 1.75, N 40.46; Found: C 20.87, H 1.81, N 39.61.

### 3.5. 1-Amino-3-nitro-1,2,4-triazole (1n)

NaOH (72 g, 1,800 mmol) was added to a solution of KH_2_PO_4_ (230 g, 1691.18 mmol) in water (1,650 mL) at room temperature, resulting in the buffered solution of KH_2_PO_4_/K_2_HPO_4_. 3-Nitro-1*H*-1,2,4-triazole (28.50 g, 250 mmol) was added to the buffered solution of KH_2_PO_4_/K_2_HPO_4_ (170 mL) at room temperature and was allowed to stir till clarified. Then HOSA (90 g, 800 mmol) was added to the solution at 80 °C and was allowed to stir for 8 h. The resultant solution was extracted with ethyl acetate. The organic phase was dried over magnesium sulfate and the solvent was removed in vacuum. The solid was purified by column chromatography (ethyl acetate/chloroform = 1:1), yielding **1n** (5.8 g, 18%) as colorless crystals. DSC (5 °C·min^−1^): 137 °C (T_d_); ^1^H-NMR (DMSO-*d_6_*) δ = 7.16 (s, 2H), 8.71 (s, 1H) ppm; ^13^C-NMR (DMSO-*d_6_*) δ = 135.0 (s, 1C) ppm; ^15^N-NMR (DMSO-*d_6_*) δ = −298.1, −151.8, −139.0, −84.0, −27.8 ppm; IR = 3331, 3207, 3134, 2974, 2027, 1799, 1648, 1556, 1508, 1458, 1414, 1311, 1194, 1032, 1016, 903, 835, 731, 663 cm^−1^; MS (EI) *m/z*: 129 (M^+^); Elemental analysis calcd (%) for (C_2_H_3_N_5_O_2_): C 18.61, N 54.26, H 2.34; found: C 18.99, N 54.63, H 2.59.

### 3.6. 1-Amino-3,5-dinitropyrazole **2n**

Freshly prepared pulverized O-p-tolylsulphonylacetohydroximate (0.8 g, 3.11 mmol) was added to 75% perchloric acid (7.5 mL) at room temperature and stirred under ambient conditions for 2 h. The now-free tosylhydroxylamine suspension was poured into 80 ml of ice/water slurry and after the ice was melted, the mixture was extracted with dichloromethane (7 × 10 mL). The combined dichloromethane extracts were dried over sodium sulfate and then added in one portion to a solution of ammonium dinitropyrazole (0.50 g, 2.84 mmol) in acetonitrile 250 mL. The solution was stirred for two days at ambient temperature, evaporated to dryness, and resuspended in ethyl acetate. The suspension was filtered and the filtrate evaporated and purified by silica chromatography using dichloromethane/hexane (2:1) as the eluent, yielding 0.246 g (50%) of 1-amino-3,5-dinitro-pyrazole. DSC (5 °C·min^−1^): 112 °C (T_d_); ^1^H-NMR (DMSO-*d_6_*) δ = 7.88 (s, 2H), 8.02 (s, 1H) ppm; ^13^C-NMR (DMSO-*d_6_*) δ = 101.7 (s, 1C) ppm; ^15^N-NMR (DMSO-*d_6_*) δ = −282.0, −160.5, −78.0, −29.4, −23.5 ppm; IR = 3356, 3289, 2027, 1691, 1567, 1556, 1507, 1468, 1396, 1358, 1335, 1290, 1133, 1097, 1012, 928, 845, 817, 741, 728, 619 cm^−1^; MS (ESI) *m/z*: 172 [M-1]^+^; Elemental analysis calcd (%) for (C_3_H_3_N_5_O_4_): C 20.82, N 40.46, H 1.75; found: C 21.01, N 40.25, H 1.93.

## 4. Conclusions

Two *C*-aminonitroazoles and the corresponding *N*-aminonitroazoles have been synthesized and fully characterized by means of vibrational and multi-nuclear NMR spectroscopy, mass spectrometry, and differential scanning calorimetry. 5-Amino-3-nitro-1*H*-1,2,4-triazole (**1c**) was successfully prepared by one-step amination reaction of 3-nitro-1*H*-1,2,4-triazole with 1,1,1-trimethylhydrazinium iodide. In comparison with previous synthetic routes of **1c**, the route is more simple and straightforward. In addition, we found that when a buffered solution of KH_2_PO_4_/K_2_HPO_4_ was used as solvent, HOSA can successfully aminate the electron-poor compound 3-nitro-1*H*-1,2,4-triazole to give the corresponding product **1n**. X-ray crystallographic measurements were performed and delivered the correlation of configuration relationships with densities and impact sensitivities. Among them, two new compounds (**1n** and **2n)** exhibit excellent impact sensitivities (IS > 40 J), high detonation performance and may serve as a promising alternative to some known explosives such as TNT.

Based on the data and crystal structures in this work and available in the literature, the regularity of *N*-amino and *C*-amino group affecting energetic properties has been developed as follows (Table 2): (i) the incorporation of an *N*-amino group into nitroazole ring can improve nitrogen content, heat of formation, and impact sensitivity. At the same time, it can also decrease density, oxygen balance, detonation velocity and pressure; (ii) the incorporation of a *C*-amino can increase density, nitrogen content, detonation velocity and pressure, while concomitantly decreasing heat of formation. Therefore, the introduction of the *C*-amino into nitroazole ring could contribute to the design of new high performance (high density and high detonation property) compounds. However, the addition of the *N*-amino into nitroazole ring is advantageous to the design of energetic compounds containing high heat of formation. We anticipate the regularity is highly useful for the design and synthesis of novel high-energy-density materials (HEDM).

**Table 2:** The regularity of *N*-amino and *C*-amino groups affecting energetic properties.

Group	N	Ω	*D*	HOF	P	D
*N*-amino	↑	↓	↓	↑	↓	↓
*C*-amino	↑	↓	↑	↓	↑	↑

↑: Increase; ↓: Decrease.

## Supplementary Materials

Supplementary materials can be accessed at: http://www.mdpi.com/1420-3049/19/1/896/s1.
