# Supplementary material for: Amination of Nitroazoles — A Comparative Study of Structural and Energetic Properties
Source: Molecules. 2014 Jan 14;19(1):896–910. doi: 10.3390/molecules19010896 (PMC6272014; doi:10.3390/molecules19010896)
Supplement: Supplementary file 1 [file molecules-19-00896-s001.pdf]

Supplemantary Materials

Figure S1. DSC and TG spectra of **1** (**a**), **1n** (**b**), **2n** (**c**) and **1c** (**d**).

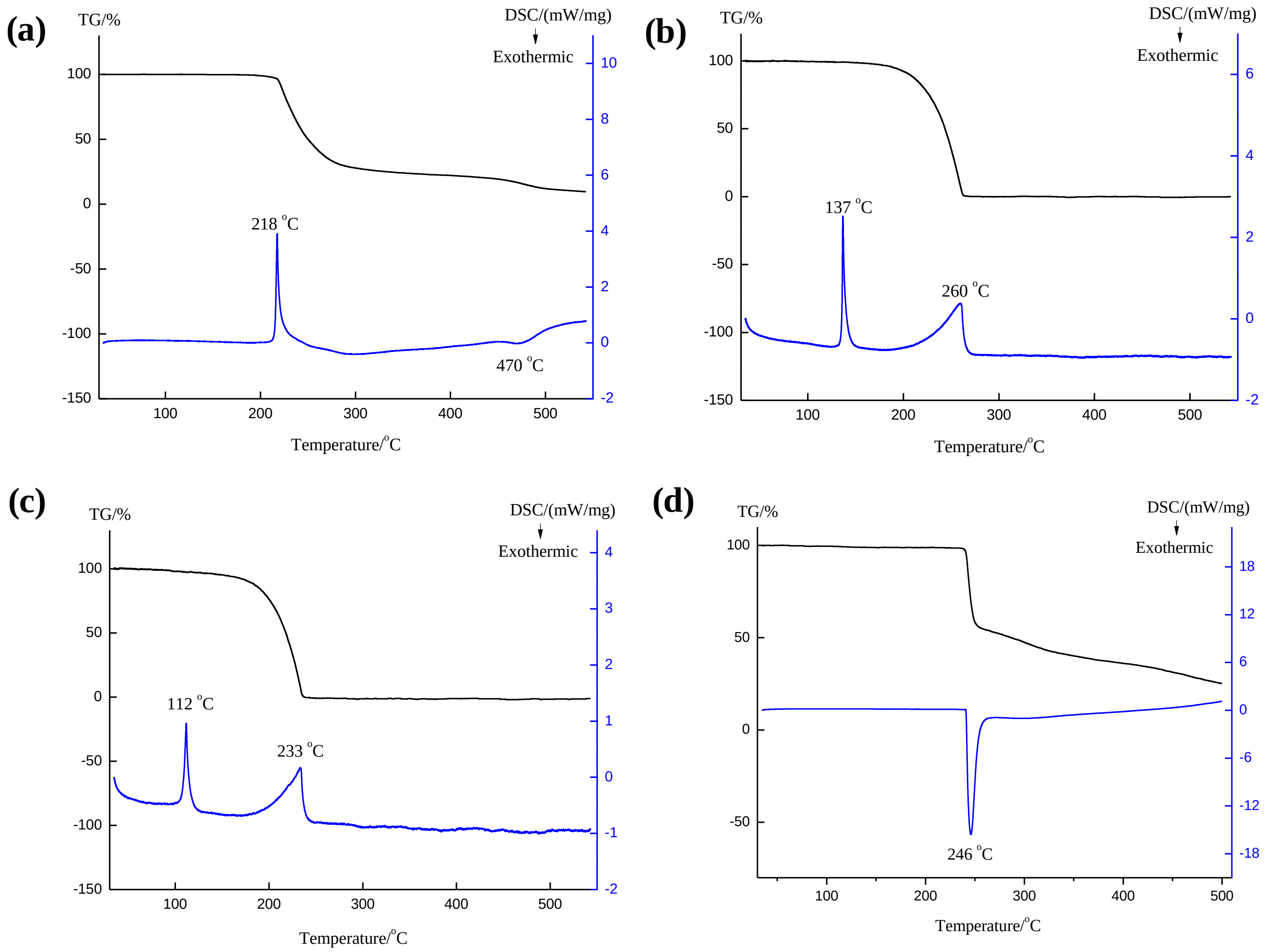

Figure S2. DSC spectra of **1**, **1n** and **1c**.

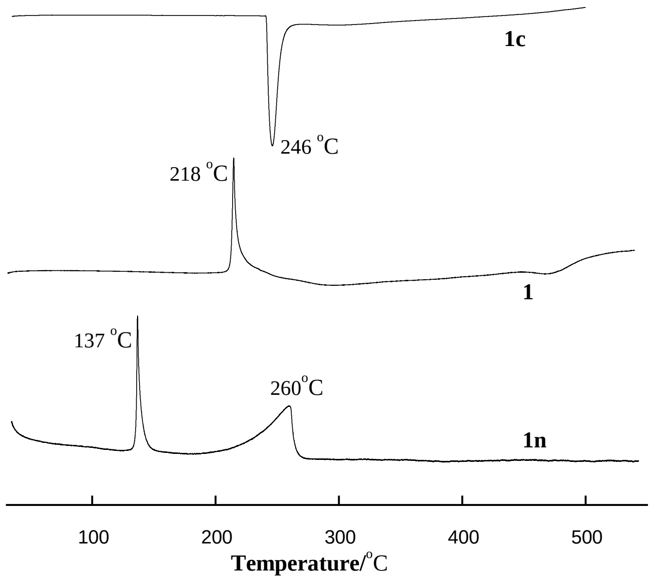

Figure S3. DSC spectra of **2**, **2n** and **2c**.

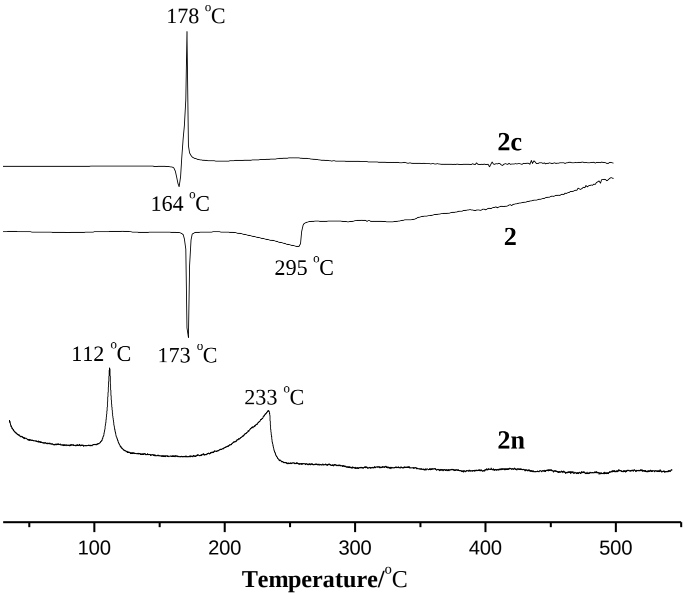

Table S1. Selected bond lengths (Å) and bond angles (°) in crystal for **1c** [1] and **1n**.

Bonds	1c (Å)	1n (Å)	Bonds	1c (Å)	1n (Å)
N1-C2	1.344	1.337	N5-O5A	1.221	1.226
N2-C2	1.309	1.315	N5-O5B	1.226	1.226
N2-N3	1.369	1.351	N3-N4		1.398
C1-N3	1.350	1.334	C1-N4	1.341	
C1-N1	1.334	1.328	C2-N5	1.447	1.452
**Bond angles**	**1c (°)**	**1n (°)**	**Bond angles**	**1c (°)**	**1n (°)**
N1-C2-N2	118.3	117.9	O5A-N5-O5B	124.1	125.8
C2-N2-N3	100.1	99.8	N2-N3-N4		122.5
C1-N3-N2	110.6	111.2	N3-N4-H4A		104.1
N1-C1-N3	109.5	109.8	N3-N4-H4B		105.0
C1-N1-C2	101.5	101.2	H4A-N4-H4B	121	112.5
N1-C2-N5	120.7	121.6	N1-C1-H1		125.1
C2-N5-O5A	117.7	117.0	N3-C1-H1		125.1
N2-C2-N5	212.0	120.5	N4-N3-C1		126.3
C2-N5-O5B	118.2	117.2			

Table S2. Selected bond lengths (Å) and bond angles (°) in crystal for **2c** [2] and **2n**.

Bonds	2c (Å)	2n (Å)	Bonds	2c (Å)	2n (Å)
N5-O5A	1.234	1.227	N5-O5B	1.229	1.225
C5-N5	1.398	1.443	C5-C4	1.390	1.377
C5-N1	1.380	1.332	N2-N4		1.390
N1-N2	1.325	1.330	N2-C3	1.346	1.360
C3-C4	1.409	1.356	C3-N3	1.424	1.444
N3-O3A	1.223	1.220	N3-O3B	1.223	1.227
C4-N4	1.334				
**Bond angles**	**2c (°)**	**2n (°)**	**Bond angles**	**2c (°)**	**2n (°)**
C4-C3-N3	126.8	126.7	O5B-N5-C5	116.8	116.5
C4-C5-N5	129.1	126.9	O5B-N5-O5A	124.5	125.1
C5-C4-C3	100.9	102.3	C4-N4-H4A	120.0	
N1-C5-N5	121.8	118.9	C4-N4-H4B	120.0	
N1-N2-C3	104.4	110.8	H4A-N4-H4B	120.0	35.3
N1-C5-C4	109.1	114.2	N2-C3-C4	114.4	109.0
N2-N1-C5	111.2	103.7	N2-C3-N3	118.8	124.2
O3A-N3-C3	116.0	115.4	O3B-N3-O3A	124.6	124.6

Table S3. Crystallographic data and structure refinement parameters for **1n** and **2n**.

	**1n**	**2n**
Empirical formula	$C_2H_3N_5O_2$	$C_3H_3N_5O_4$
CCDC	958583	958582
Temperature/K	153(2)	180(10)
Wavelength/Å	0.71073	0.71073
Crystal system	Orthorhombic	Orthorhombic
Space group	$Pna2_1$	$P2_12_12_1$
a /Å	9.919(6)	5.4902(7)
b /Å	9.880(6)	9.7958(12)
c /Å	5.152(3)	11.7902(17)
α /°	90	90
β /°	90	90
γ /°	90	90
V /Å ^{3}	504.9(5)	634.09(15)
Z	4	4
ρ /(g cm^{-3})	1.698	1.813
μ /mm^{-1}	0.149	0.167
F(000)	264	352
Crystal size/mm	0.41 × 0.33 × 0.25	0.2 × 0.1 × 0.1
θ / °	2.91~30.03	3.45~25.99
Limiting indices	$-13 \le h \le 13$<br>$-9 \le k \le 13$<br>$-7 \le l \le 7$	$-6 \le h \le 5$<br>$-12 \le k \le 9$<br>$-14 \le l \le 13$
Reflections Collected	4096	1869
Independent reflections	807	1195
R_{int}	0.0426	0.0263
Final R indices [I > 2σ (I)]	R_1 = 0.0313, wR_2 = 0.0663	R_1 = 0.0474, wR_2 = 0.1135
Final R indices (all data)	R_1 = 0.0348, wR_2 = 0.0680	R_1 = 0.0587, wR_2 = 0.1245

Heat of formation

Isodesmic reaction, in which numbers of electron pairs and chemical bond types are conserved, has been employed very successfully to give heat of formation more accurate than semi-empirical calculation [3]. Based on the optimized structures, the total energy (E_0) and thermodynamic parameters, including zero point energy (ZPE) and thermal correction to enthalpy (H_T), were obtained at the B3LYP/6-311++g(d, p) level.

For the isodesmic reaction (Scheme S1), heat of reaction (ΔH_{298K}) can be calculated from the following Equation (1):

$$H_{298\ K} = \Delta H_{f,P} - \Delta H_{f,R} \quad (1)$$

where $\Delta H_{f,R}$ and $\Delta H_{f,P}$ are the heats of formation for reactants and products at 298.15 K, respectively. Meanwhile, $\Delta H_{298\ K}$ can also be calculated using the following Equation (2):

$$\Delta H_{298\ K} = \Delta E_{298\ K} + \Delta(PV) = \Delta E_0 + \Delta ZPE + \Delta HT + \Delta(nRT) \quad (2)$$

Where ΔE0 is the change in total energy between the products and the reactants at 0 K; ΔZPE is the difference between the zero-point energies of the products and the reactants; ΔHT is thermal correction from 0 K to 298.15 K. Since there is no change in number of total molecules, Δ(PV) = Δ(nRT) =0. Therefore, the heat of formation can be figured out according to ΔH298 and heats of formation of other reactants and products. Fortunately, these data can be acquired from the literature and handbook facilely.

Scheme S1. Isodesmic reactions.

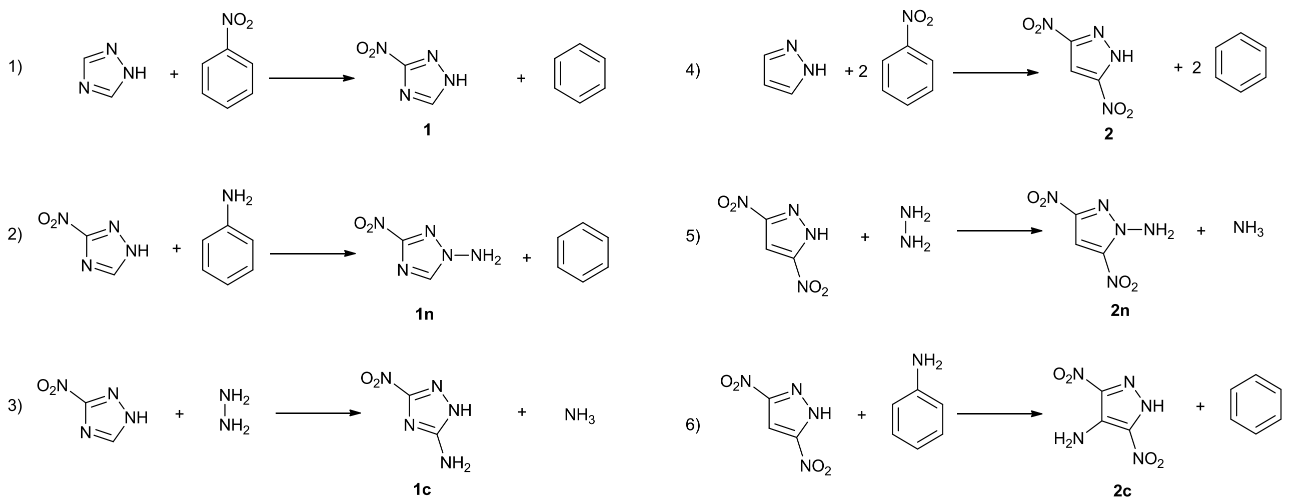

Table S4. Calculated total energy (E_0), zero-point energy (ZPE), thermal correction (HT), and enthalpy of formation (HOF) of azole-based compounds and reference compounds.

Compd.	E_0/a.u.	ZPE (kJ/mol)	HT (kJ/mol)	HOF (kJ/mol)
Nitrobenzene [4]	−436.750585	271.97	20.26	68.53
Benzene [5]	−232.248647	264.51	14.00	82.9
Aniline [6]	−287.601761	308.21	17.66	87
1H-1,2,4-triazole [7]	−242.249274	157.47	11.84	192.7
1H-pyrazole [8]	−226.198601	187.47	12.35	177.4
Ammonia [9]	−56.547948	90.67	9.99	45.94
Hydrazine [10]	−111.856446	140.43	11.02	95.35
**1**	−446.736094	163.84	18.08	216.9
**1n**	−502.059252	207.93	19.21	217.9
**1c**	−502.096652	207.60	22.04	201.8
**2**	−635.183613	201.20	24.93	128.5
**2n**	−690.433312	244.42	28.58	144.4
**2c**	−690.490506	245.22	28.68	96.3

Figure S4. ^{1}H NMR spectrum (400 MHz) of **1c** in DMSO-d_6 at 25 °C.

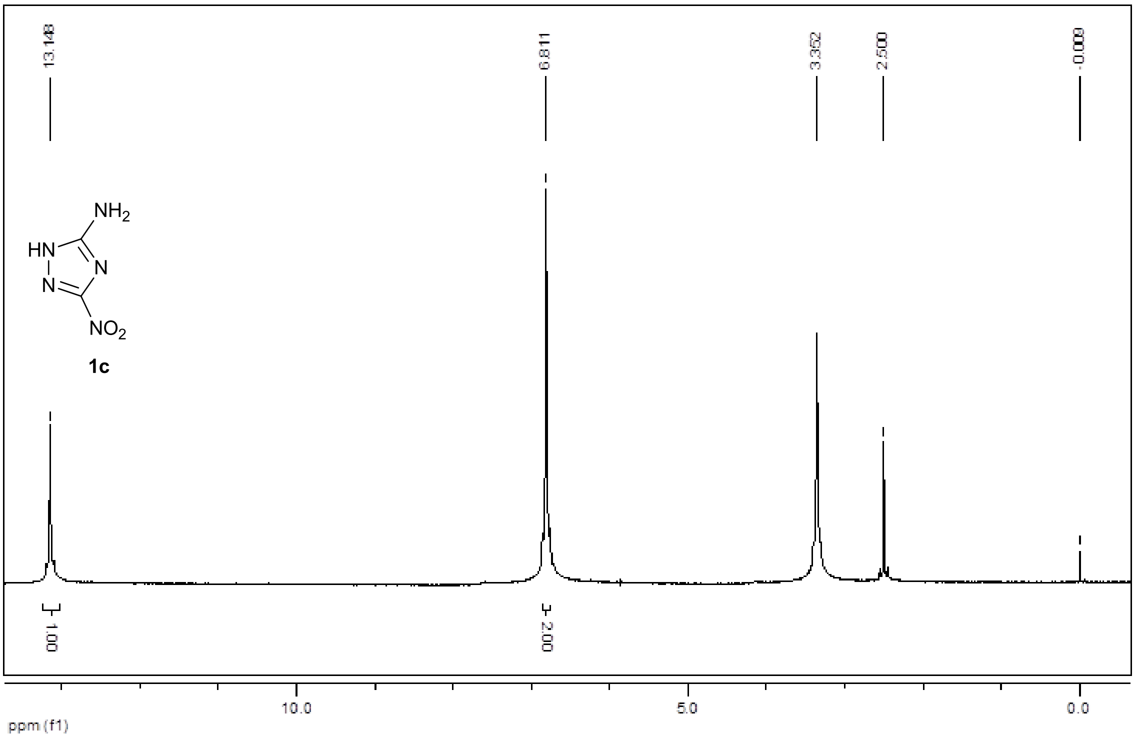

Figure S5. ^{13}C NMR spectrum (100 MHz) of **1c** in DMSO-d_6 at 25 °C.

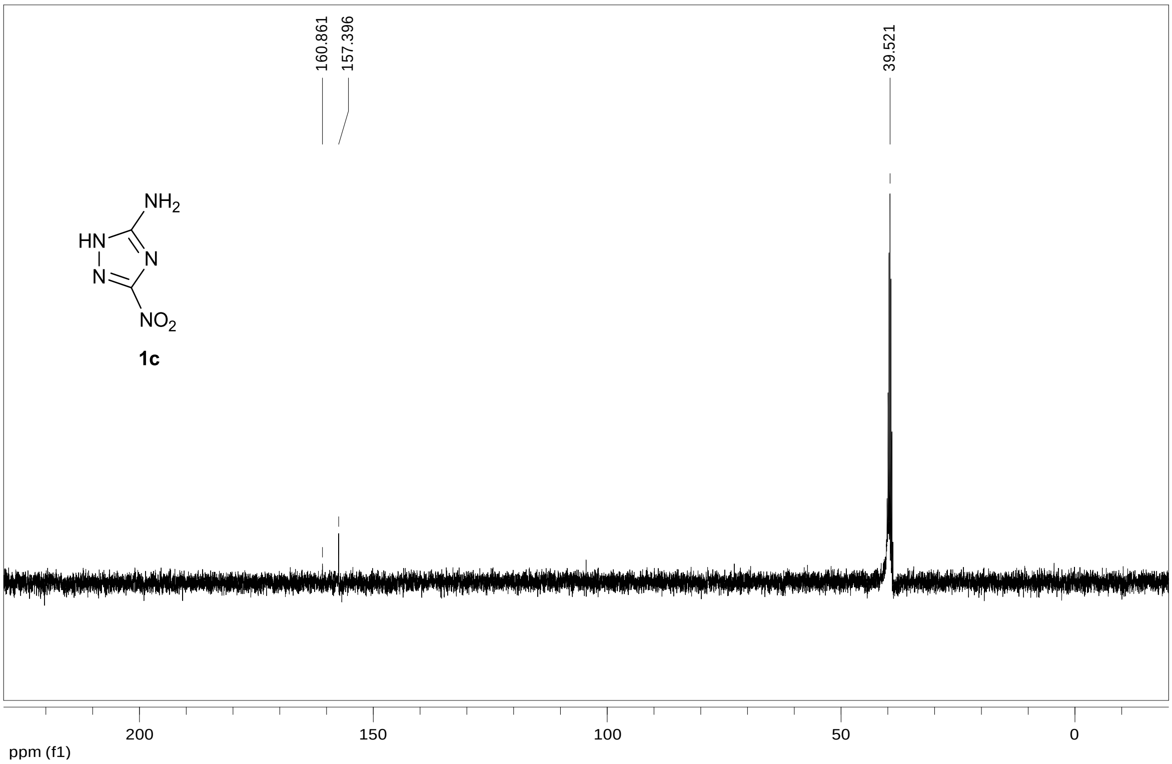

Figure S6. ESI-MS spectrum of **1c**.

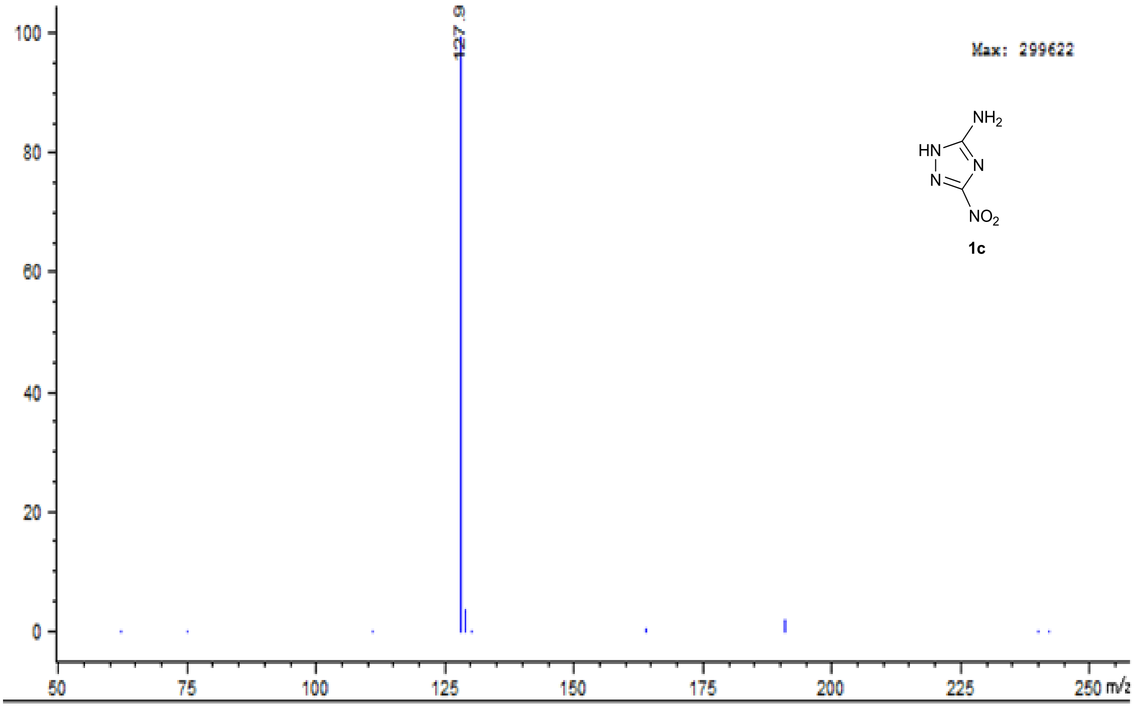

Figure S7. ^{1}H NMR spectrum (400 MHz) of **2** in DMSO-d_6 at 25 ℃.

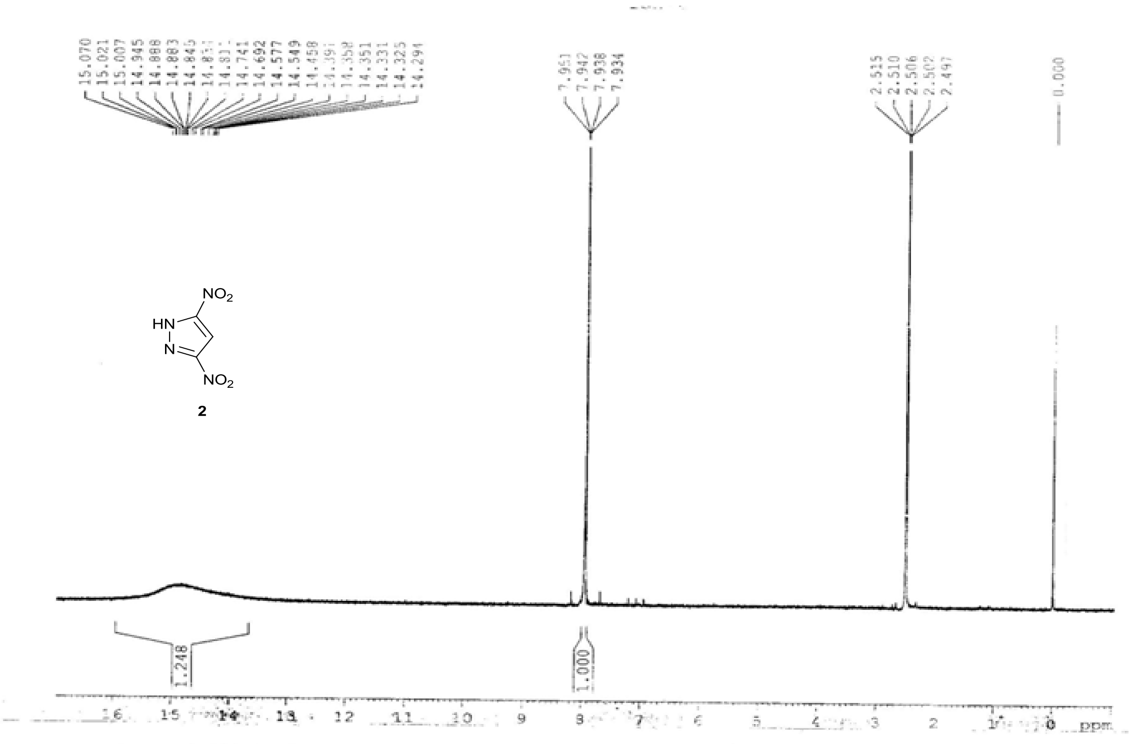

Figure S8. EI-MS spectrum of **2**. Provide higher resolution figure.

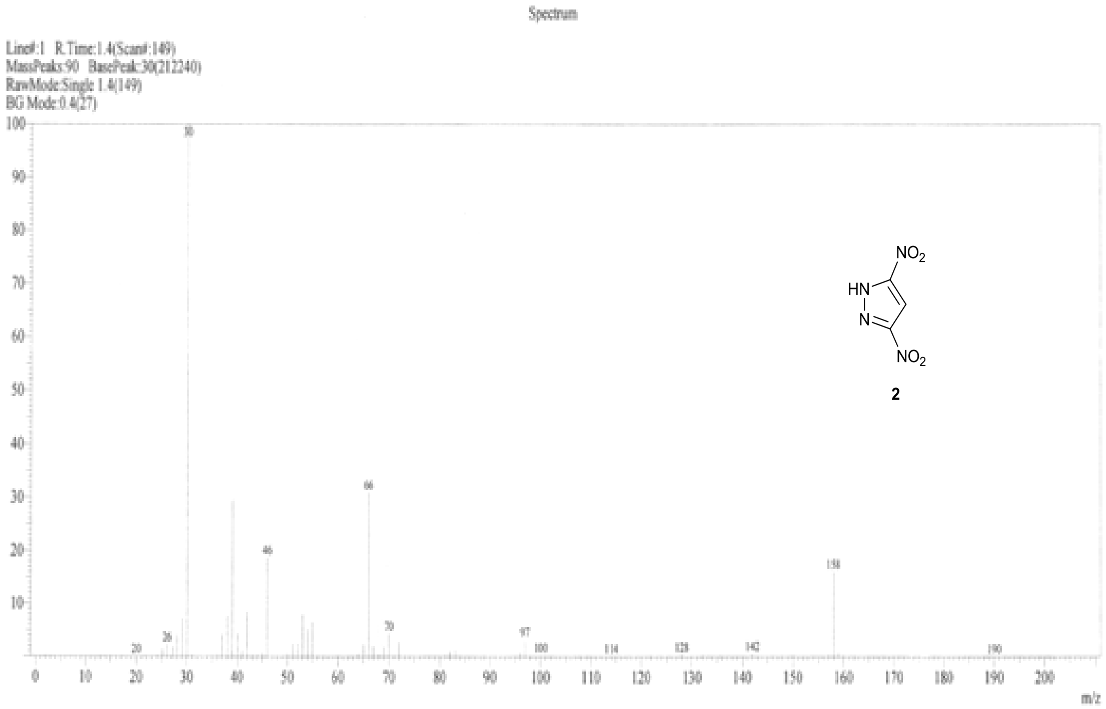

Figure S9. ^{1}H NMR spectrum (400 MHz) of **2c** in DMSO-d_6 at 25 °C.

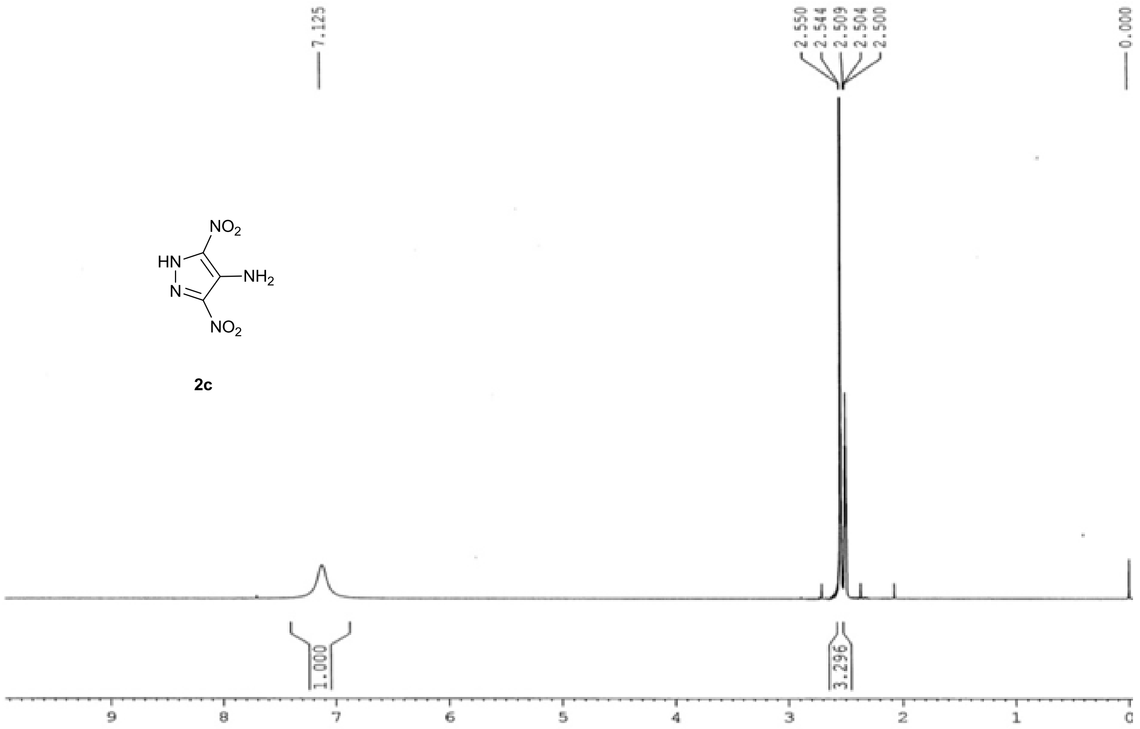

Figure S10. ESI-MS spectrum of **2c**.

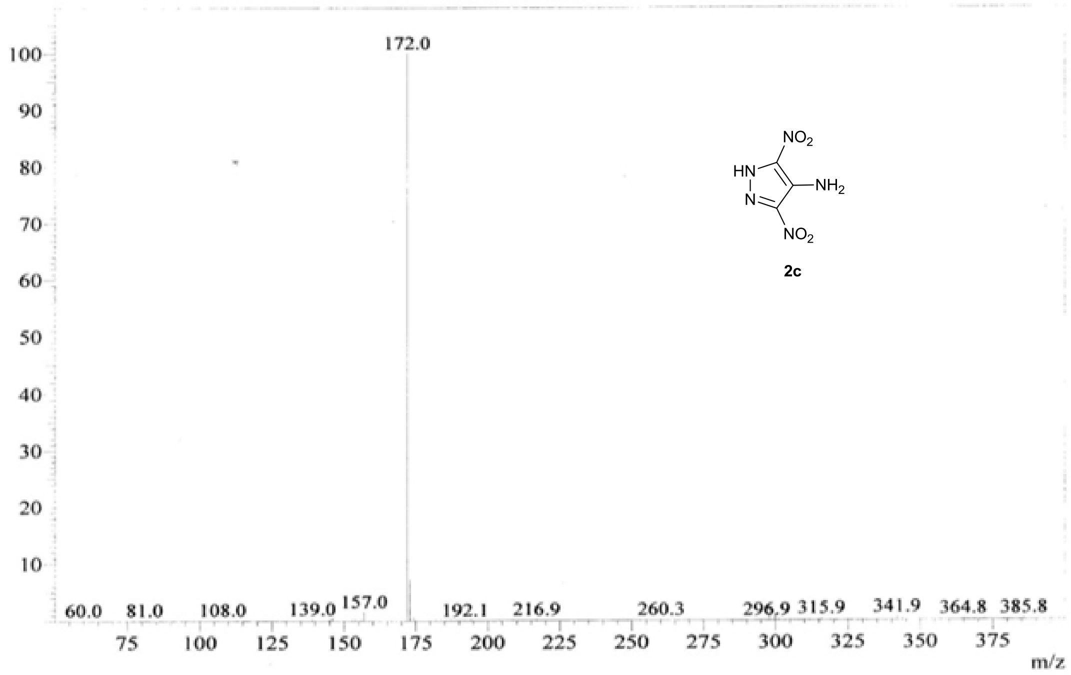

Figure S11. ^{1}H NMR spectrum (400 MHz) of **1n** in DMSO-d_6 at 25 °C.

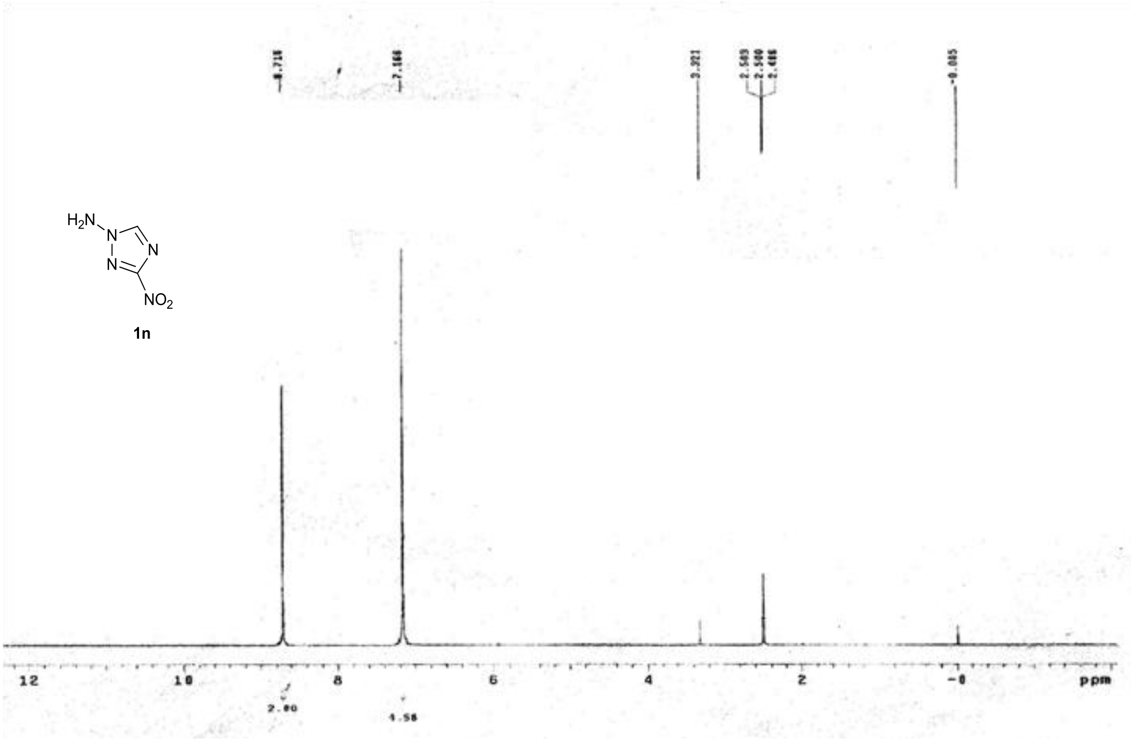

Figure S12. ^{13}C NMR spectrum (100 MHz) of **1n** in DMSO-d_6 at 25 °C.

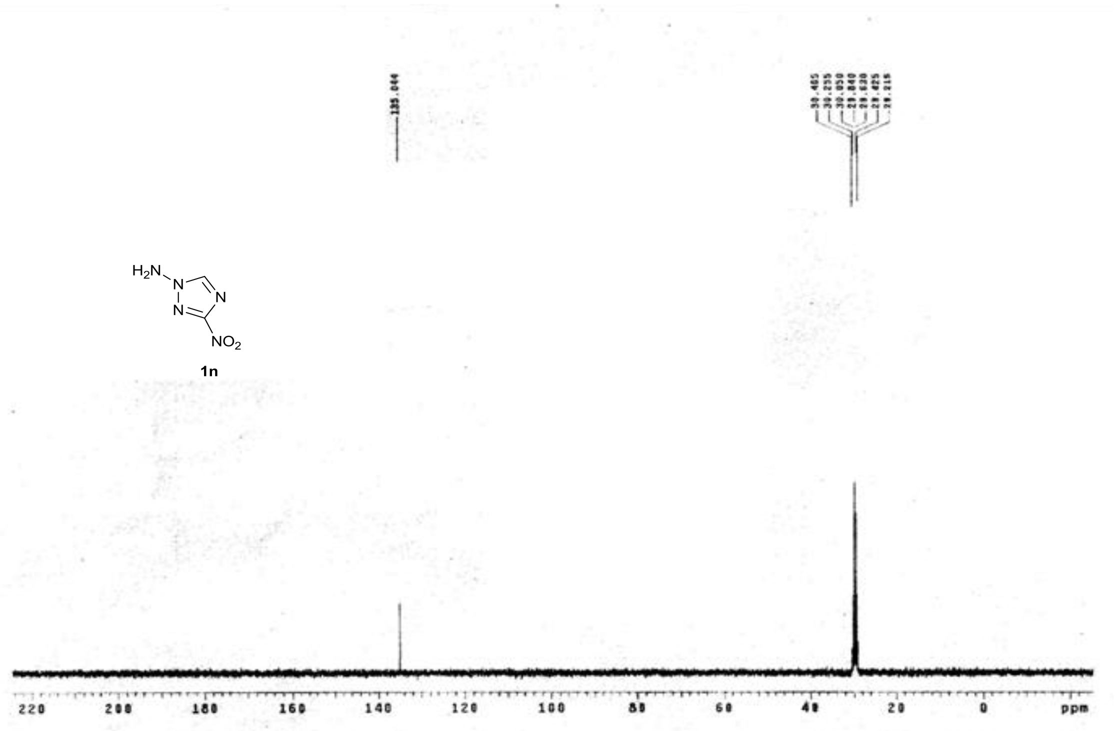

Figure S13. EI-MS spectrum of **1n**.

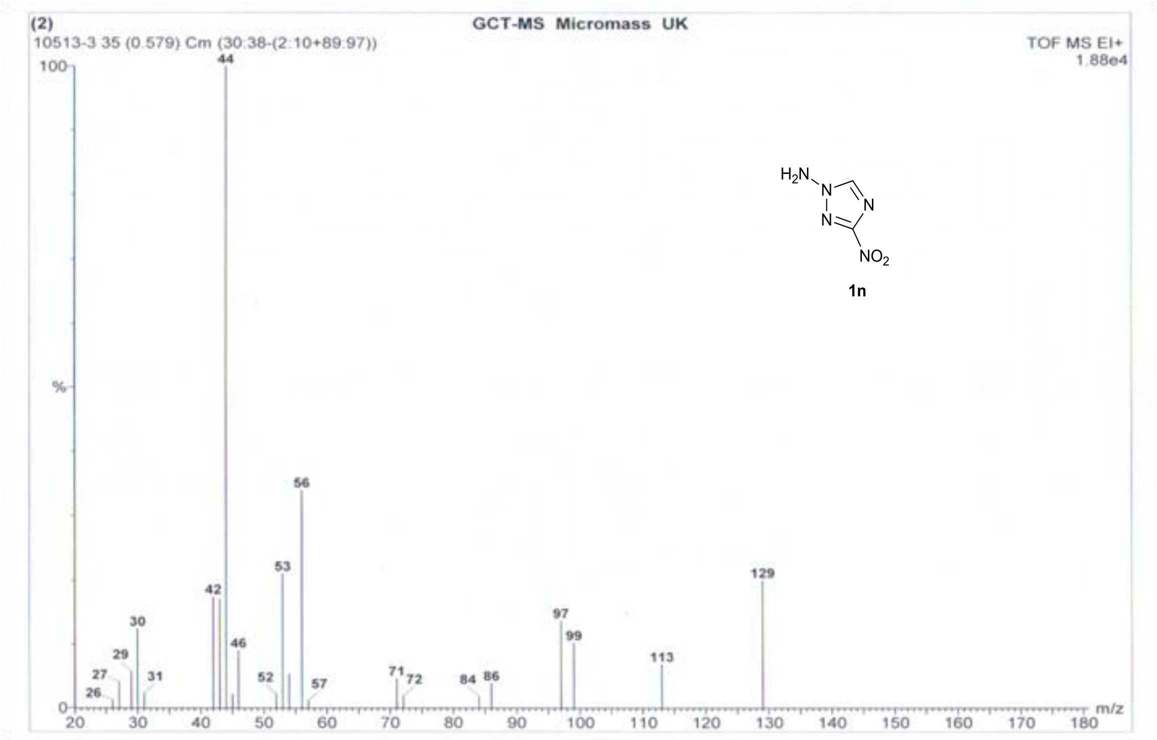

Figure S14. IR spectrum of **1n**.

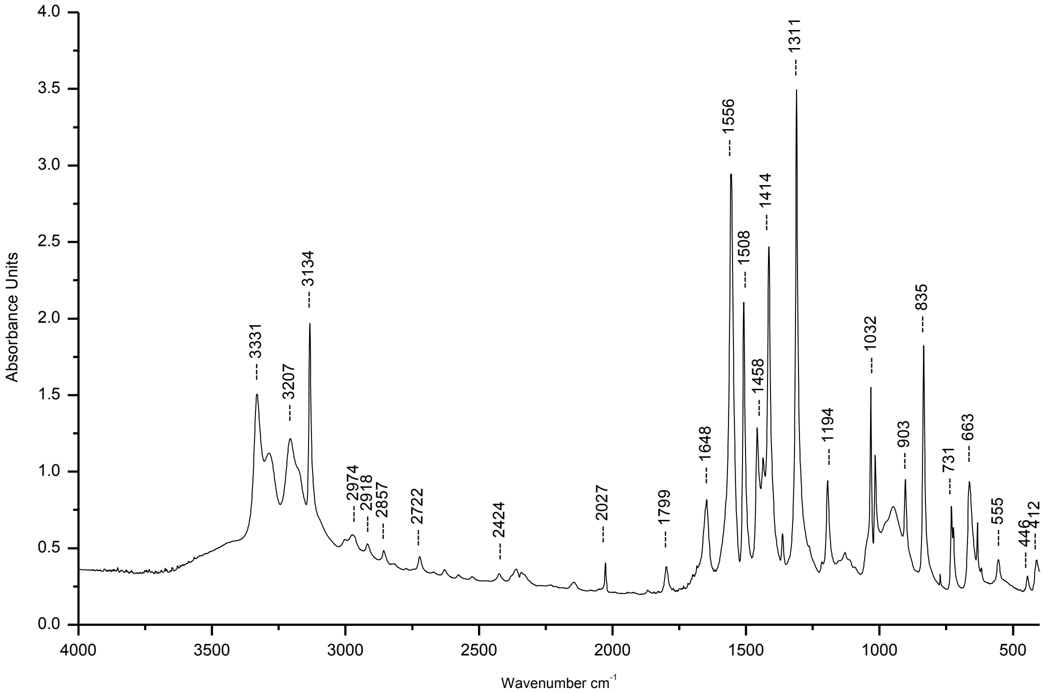

Figure S15. ^{1}H NMR spectrum (400 MHz) of **2n** in DMSO-d_6 at 25 ℃.

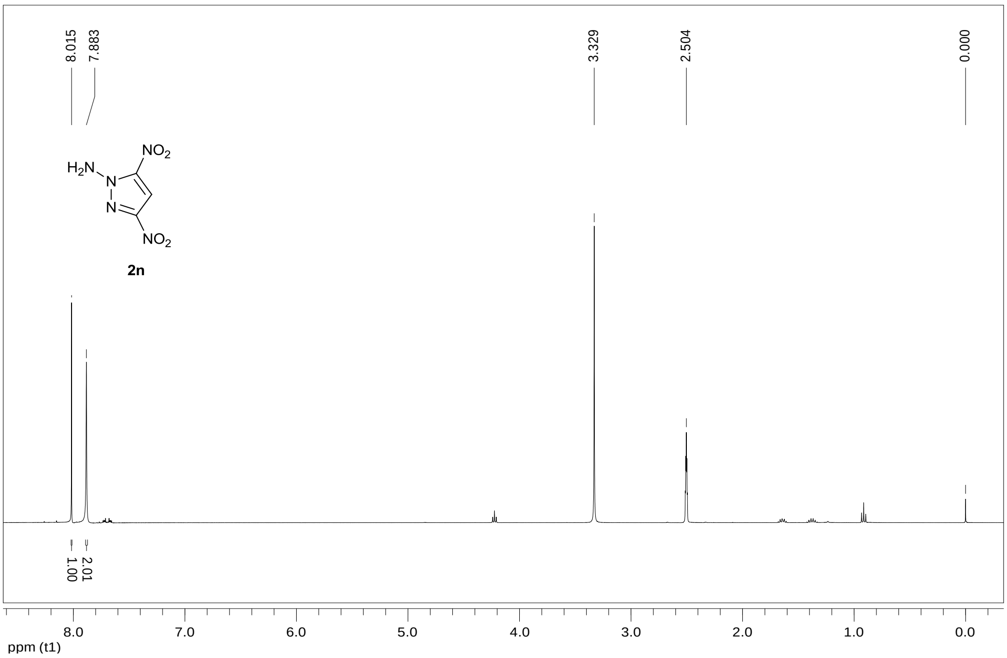

Figure S16. ^{13}C NMR spectrum (100 MHz) of **2n** in DMSO-d_6 at 25 ℃.

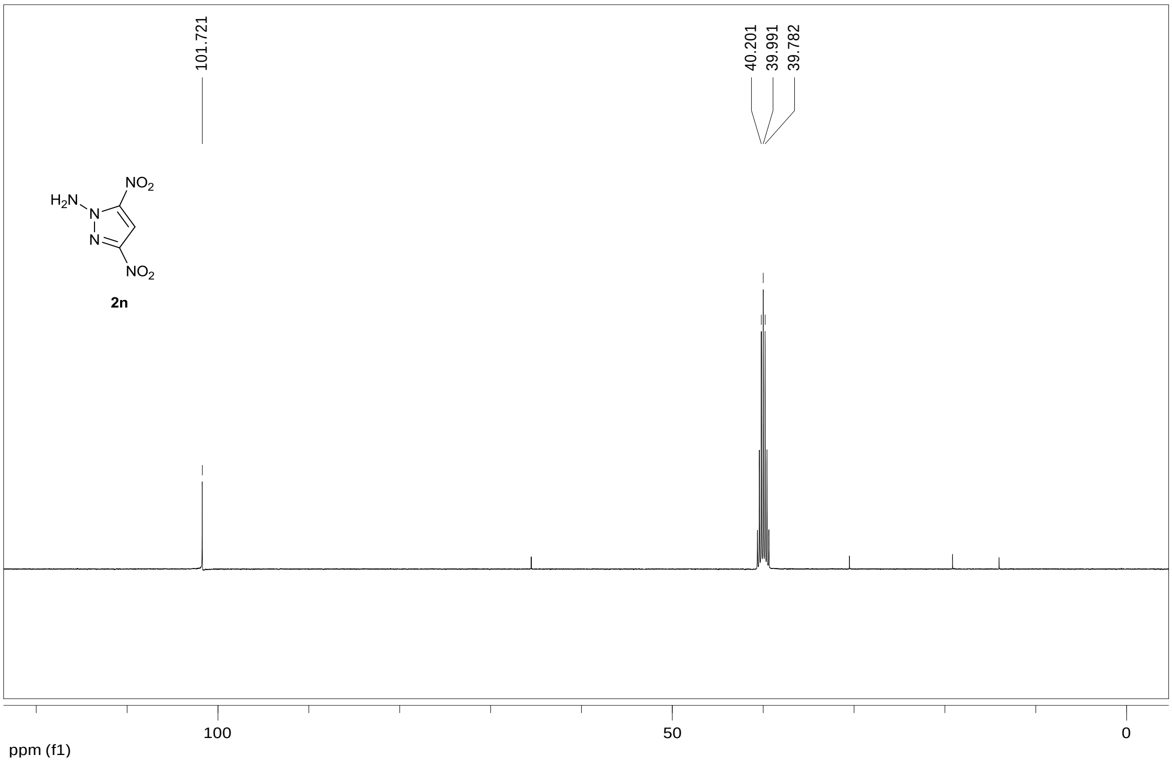

Figure S17. ESI-MS spectrum of **2n**.

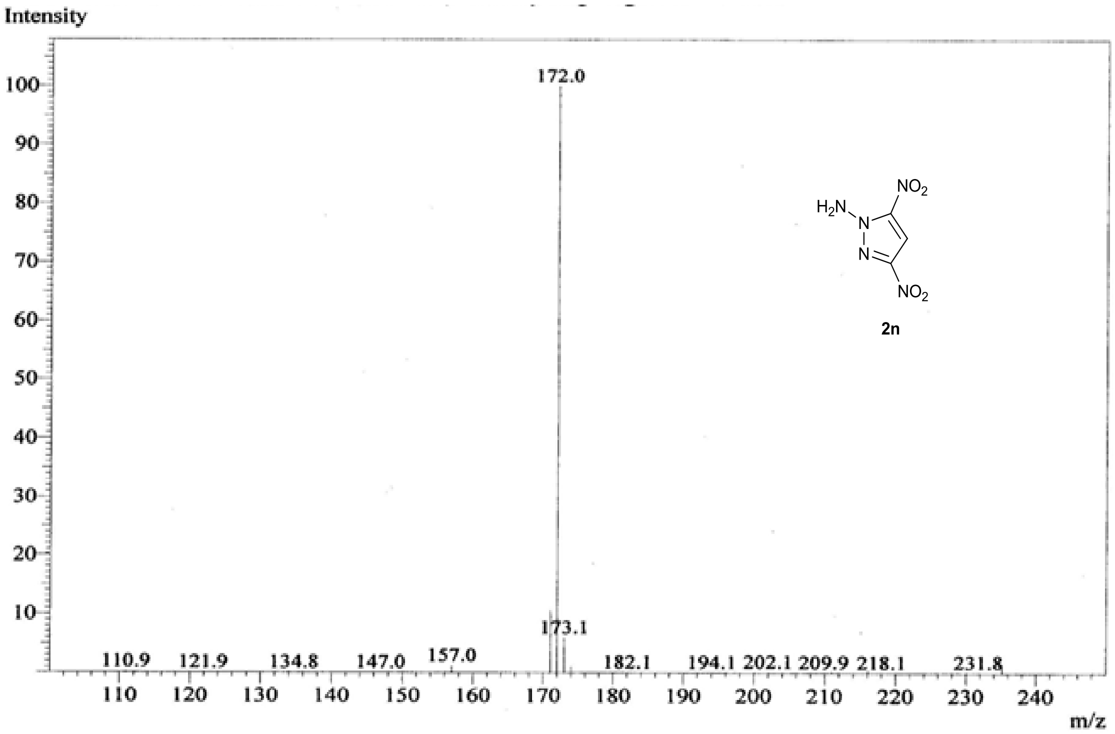

Figure S18. IR spectrum of **2n**.

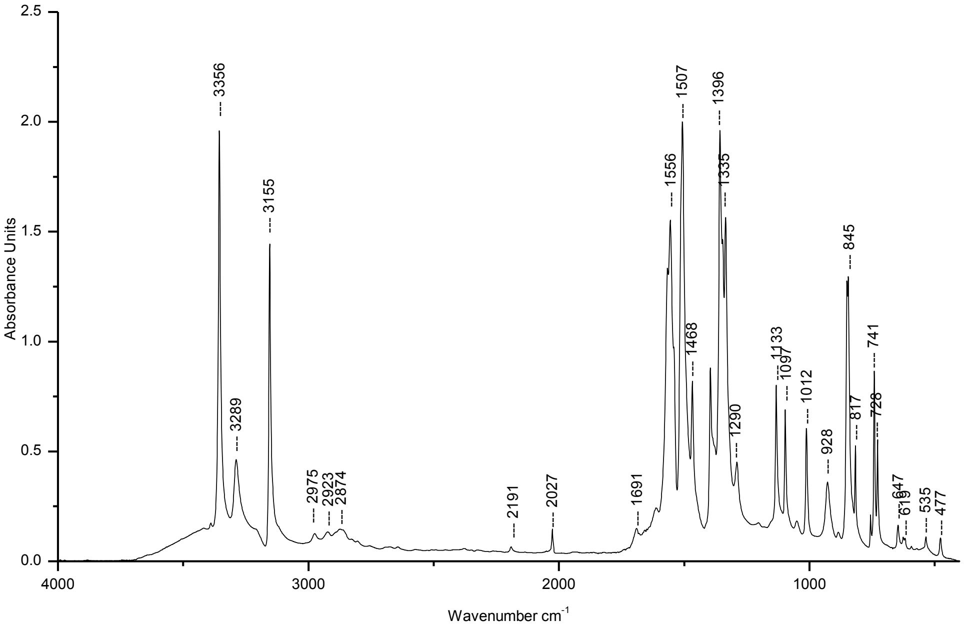

References

1. Garcia, E.; Lee, K.Y. Structure of 3-amino-5-nitro-1,2,4-triazole. *Acta Cryst.* **1992**, *C48*, 1682–1683.
2. Schmidt, R.D.; Lee, G.S.; Pagoria, P.F.; Mitchell, A.R. Synthesis of 4-amino-3,5-dinitro-1*H*-pyrazole using vicarious nucleophilic substitution of hydrogen. *J. Heterocycl. Chem.* **2001**, *38*, 1227–1230.
3. Hehre, W.J.; Radom, L.; Schleyer, P.V.R.; Pople, J.A. *Ab Initio Molecular Orbital Theory*; Wiley: New York, NY, USA, 1986.
4. Lebedeva, N.D.; Katin, Y.A.; Akhmedova, G.Y. Standard enthalpy of formation of nitrobenzene. *Russ. J. Phys. Chem.* **1971**, *45*, 1192–1193.
5. Roux, M.V.; Temprado, M.; Chickos, J.S.; Nagano, Y. Critically evaluated thermochemical properties of polycyclic aromatic hydrocarbons. *J. Phys. Chem. Ref. Data* **2008**, *37*, 1855–1996.
6. Hatton, W.E.; Hildenbrand, D.L.; Sinke, G.C.; Stull, D.R. Chemical thermodynamic properties of aniline. *J. Chem. Eng. Data* **1962**, *7*, 229–231.
7. Jimenez, P.; Roux, M.V.; Turrion, C. Thermochemical properties of *N*-heterocyclic compounds II. Enthalpies of combustion, vapour pressures, enthalpies of sublimation, and enthalpies of formation of 1,2,4-triazole and benzotriazole. *J. Chem. Thermodyn.* **1989**, *21*, 759–764.
8. Zaheeruddin, M.; Lodhi, Z.H. Enthalpies of formation of some cyclic compounds. *Phys. Chem.* **1991**, *10*, 111–118.
9. Cox, J.D.; Wagman, D.D.; Medvedev, V.A. *Codata Key Values for Thermodynamics*; Hemisphere Publishing Corp: New York, NY, USA, 1984.
10. Chase, M.W. NIST-JANAF themochemical tables. *J. Phys. Chem. Ref. Data* **1998**, *9*, 1948–1951.
